# A Presentation of Strawberry Gingivitis as the Initial Presenting Symptom of Recurrence of Granulomatosis With Polyangiitis

**DOI:** 10.7759/cureus.80945

**Published:** 2025-03-21

**Authors:** Marissa N McPhail, Michael Wu, Hassaan Wajeeh, Christina Thymalil, Rohit Muralidhar, Marc M Kesselman

**Affiliations:** 1 Osteopathic Medicine, Nova Southeastern University Dr. Kiran C. Patel College of Osteopathic Medicine, Fort Lauderdale, USA; 2 Rheumatology, Nova Southeastern University Dr. Kiran C. Patel College of Osteopathic Medicine, Fort Lauderdale, USA

**Keywords:** gingival depigmentation, granulomatosis with polyangiitis (gpa), internal medicine and rheumatology, strawberry gingivitis, wegner’s granulomatosis

## Abstract

Granulomatosis with polyangiitis (GPA) is a rare rheumatologic small vessel vasculitis that affects multiple systems, most commonly the sinuses, lungs, and kidneys. In this case report, we detail a case of a 48-year-old male with a past medical history of GPA diagnosed two years ago and no other significant medical history. He presented with isolated gingival edema and discoloration, later identified as strawberry gingivitis, which was the initial presenting symptom of his GPA relapse. The patient's symptoms had been under control since initial induction therapy with IV prednisone, cyclophosphamide, and two doses of rituximab, and he was then continued on a maintenance therapy regimen of azathioprine for two years symptom-free. Upon identification of the patient's relapse, he was treated with a three-week course of oral prednisone to be taken twice daily. His new maintenance therapy regimen included avacopan, azathioprine, and prednisone. Since initiating this therapy, he has remained symptom-free with no new manifestations or signs of relapse.

## Introduction

Granulomatosis with polyangiitis (GPA) is a rare multisystem disease with an estimated prevalence of three in 100,000 inhabitants with a mean age of onset of 45 years old [1]. GPA is identified as an idiopathic necrotizing small vessel vasculitis commonly affecting the sinuses, lungs, and kidneys [1]. Patients may present differently depending on the extent of the disease and the body system affected. GPA can affect a mirage of body systems; however, GPA is commonly characterized by a triad of respiratory symptoms, systemic vasculitis, and kidney involvement [2]. The respiratory symptomatology can present as upper respiratory tract symptoms (sinusitis, stuffiness, and saddle deformity) and/or lower respiratory tract symptoms (cough, dyspnea, hemoptysis, and pulmonary nodules) [3]. Initial renal involvement is uncommon, but it emerges in approximately 80% of patients within a two-year timeframe, often appearing as rapidly progressive crescentic glomerulonephritis [3]. Other systems that can be affected by GPA include eyes (scleritis and conjunctivitis), ears (hearing loss), skin (purpura of lower extremities), nervous (peripheral neuropathies), and musculoskeletal (arthralgia and myalgia) [3]. Laboratory evaluation is imperative in the diagnosis of GPA, with the hallmark finding being a positive proteinase 3- antineutrophil cytoplasmic antibody (PR3-ANCA) or cytoplasmic antineutrophil cytoplasmic antibodies (c-ANCA). Although these are not required for diagnosis, they are highly characteristic of the disease [3]. Diagnosis of GPA is highly likely in the presence of two of the four American College of Rheumatology 1990 criteria (ACR) [3]. These include abnormal urinary sediment, abnormal chest radiographs, oral ulcers or nasal discharge, and granulomatous inflammation on biopsy [4].

Hyperplastic gingivitis, also known as strawberry gingivitis, is an oral physical exam finding that earned its name by its unique characteristic of exophytic gingival swellings with petechial hemorrhages [5]. Gingivitis can involve the whole gingiva and periodontium, leading to complications causing tooth mobility and loss of teeth [6]. Biopsies of strawberry gingivitis reveal pseudoepitheliomatous hyperplasia of overlying epithelium with occasional multinucleated giant cells, numerous eosinophils, and diffuse chronic histiocyte inflammation [6]. The biopsy specimens are commonly stained with hematoxylin and eosin, as well as methamine silver nitrate and periodic acid-Schiff stain. This significant finding is important for clinicians to recognize as this can be a key finding in a clinician's effort to diagnose GPA [7]. Although the etiology of strawberry hyperplasia has only been linked to GPA, other pathological processes have been seen to cause similar-looking dental growths [8]. Some of the few identified are drug-induced gingival hyperplasia, vasculitic lesions such as hemangiomas and pyogenic granulomas, and Kaposi sarcoma [8]. With many other entities resembling the strawberry gingivitis lesion, biopsy with histologic verification is the preferred method of diagnosis [8]. To help differentiate strawberry gingivitis from other similar-looking dental lesions.

Strawberry gingivitis is a key and uncommon oral finding in GPA patients. It is also a unique finding that occurs in a region of overlap between physicians and dentists. This case presentation aims to further educate clinicians on key GPA findings from the physical exam. It also aims to show the interplay between physicians and dentists and the interdisciplinary work needed to care for GPA patients.

## Case presentation

**Figure 1:**
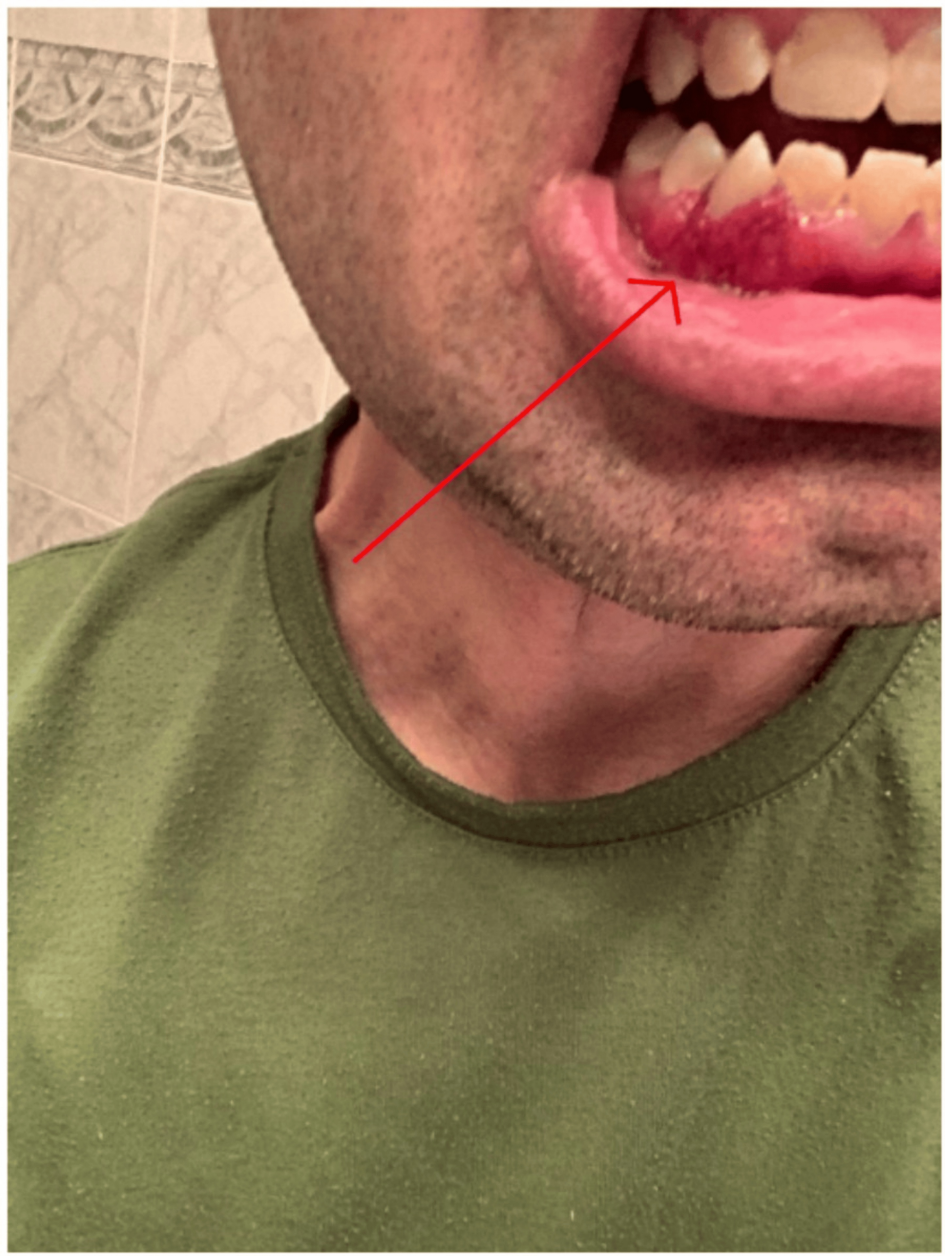
Patient's strawberry gingivitis on the lower mandible

**Figure 2:**
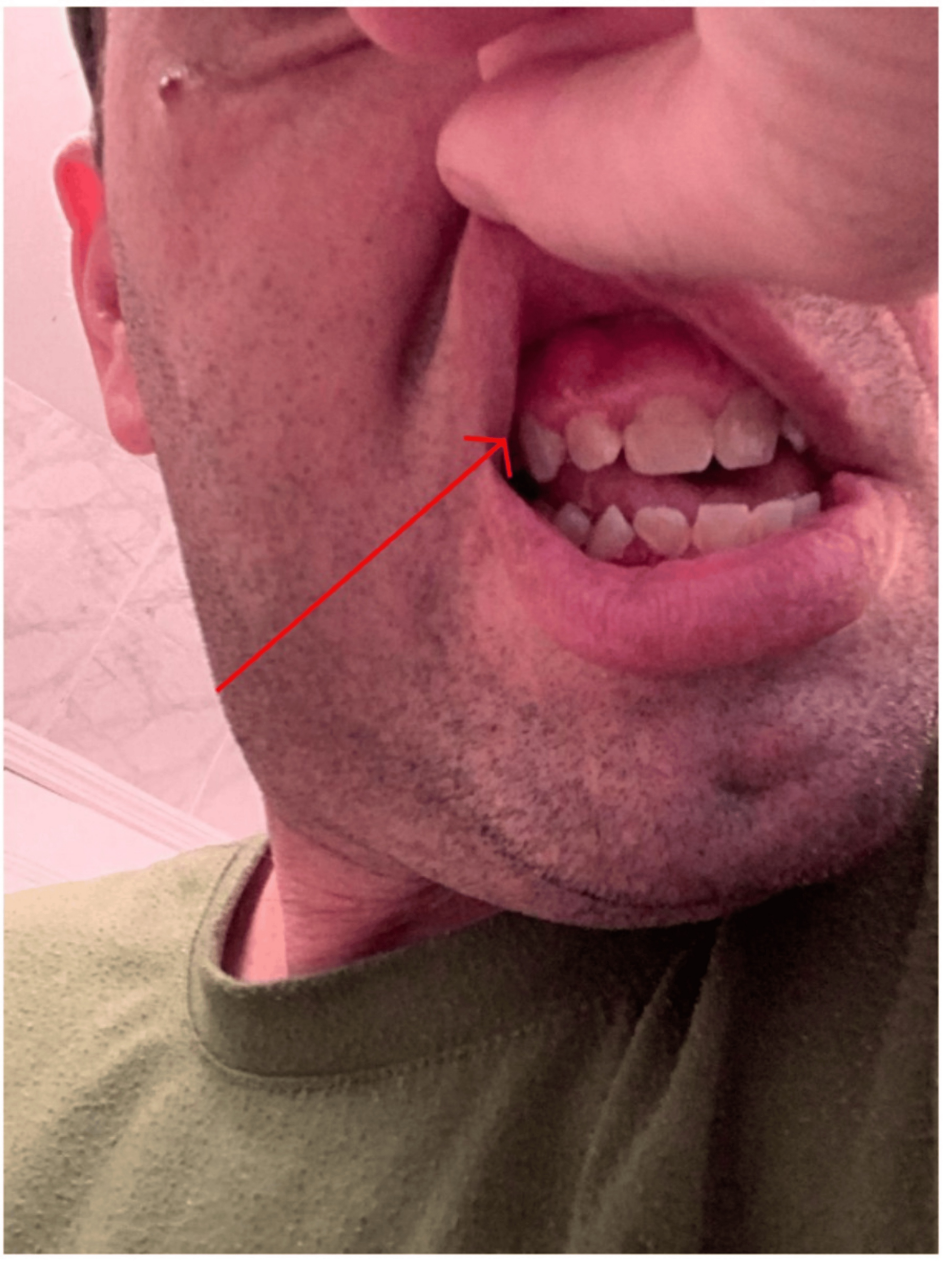
Patient's strawberry gingivitis in the upper mandible

A 48-year-old male presents to the rheumatology clinic with the complaint of erythematous gingival edema, discoloration, petechiae, and hyperplasia on both the lower gingiva as seen in Figure 1 and upper gingiva as seen in Figure 2. The patient denied any pain or bleeding from the lesions. He has a past medical history of GPA diagnosed via a prior lung biopsy for which he underwent induction therapy with a combination of IV prednisone, cyclophosphamide, and two doses of rituximab and was then continued on a maintenance therapy regimen of azathioprine for the past two years. Before this clinic encounter, the patient had been symptom-free since the initiation of his drug regimen. Aside from his isolated oral manifestations of gingival edema and discoloration, he was also found to have abnormal laboratory findings. He continued to have positive C-ANCA antibodies and MP3 markers. He also was found to have elevated inflammatory markers with a CRP of 8.0 mg/L (0 mg/L - 3 mg/L), ESR of 20 mm/hr (2 mm/hr - 10 mm/hr), and normal kidney functions with a GFR of 65 mL/min/1.73 m2 (>95 mL/min/1.73 m2) near the patient's baseline and a creatinine of 1.2 mg/dL (0.7 mg/dL to 1.3 mg/dL). Upon interviewing the patient, he denied any other symptoms aside from the oral manifestation. On physical exam, the patient was found to have clear breath sounds bilaterally, a normal heart rate and rhythm, clear sinuses, and no swollen joints or skin manifestations. Given the patient's abnormal oral manifestations and labs, a dental referral was given to the patient. The dental surgeon performed an oral biopsy with a histopathologic examination of the lesion confirming the diagnosis of strawberry gingivitis. The patient was then sent back to the rheumatology clinic, where he was diagnosed with a relapse of his GPA. He was prescribed a three-week course of oral prednisone to be taken twice daily, and a discussion of new maintenance therapy was undergone with the patient, as this was the first recurring symptom experienced by the patient since his initial diagnosis of Wegner's granulomatosis. Upon completion of the oral steroid regimen, the lesion had fully resolved, and he was free of any symptoms. A new maintenance therapy has been started for the patient consisting of avacopan, azathioprine, and prednisone. He has since been symptom-free with no new manifestations or any new signs of possible relapse.

## Discussion

ANCA-associated vasculitis (AAV) is comprised of three different necrotizing autoimmune vasculitides that affect small and medium-sized vessels [8]. The three vasculitides that make up AAV are GPA, microscopic polyangiitis (MPA), and eosinophilic granulomatosis with polyangiitis (eGPA). In AAV, neutrophils are deemed to be the predominant effector cell of pathogenesis [9]. Kessenbrock et al. discovered that ANCA-stimulated neutrophils release neutrophil extracellular traps (NETs), which can deposit and accumulate in various organs [9]. Thus, activating dendritic cells and leading to the autoimmune damage seen with AAV. While the exact mechanism of GPA is unknown, it is presumed to have a multifactorial mode of pathogenesis including the complex interaction between genetics, infection, and inflammation. The genetic component of GPA has been associated with a few different genes, including the proteinase 3 (PRTN3) gene. The main etiological factor for GPA is credited to be anti-neutrophilic cytoplasmic antibodies (ANCA) [3]. ANCA reacts with a neutrophilic enzyme, proteinase 3 (PR3), and induces degranulation of neutrophils to damage endothelial cells and increase inflammation [3]. Defective immune responses to environmental insults lead to excessive production of Th1 and Th17 cytokines, which ultimately lead to the characteristic inflammatory granulomatous vascular lesions [3]. 

Oral manifestations of GPA are a lesser-known symptom, only being reported in an estimated 6-13% of cases of GPA, with oral lesions being the initial presentation in an estimated 2% [7]. Oral ulcers are the more common oral presentation of GPA and have become a diagnostic criterion of the ACR for GPA diagnosis [4]. Strawberry gingivitis is a rare symptom of GPA that has been described a handful of times as one of the initial presenting symptoms of GPA but has not been commonly described as a symptom of GPA relapse. Strawberry gingivitis lesions may remain in the oral cavity for long periods before the classic multi-organ involvement of GPA occurs [5]. This rare oral manifestation of relapsing GPA presents a unique interplay between physicians and oral health professionals, as they may be the initial healthcare providers to identify it on routine physical exams. Timely recognition of this symptom will provide a quicker response to a relapsing GPA condition and induction of earlier maintenance therapy to reduce systemic manifestations and possible severe complications [5].

The pathophysiology behind the relapses of GPA is controversial across literature, with some studies finding lung involvement, cardiovascular involvement, persistent hematuria, and skin involvement; however, these risk factors were all not found by Tufan et al. in a single-center retrospective cohort study [10]. However, there was one risk factor that was agreed upon in several studies. The rate of relapses of GPA has been found to be strongly associated with chronic nasal carriage of *Staphylococcus aureus* (*S. aureus*) [11]. This increase in disease activity was potentially attributed to molecular mimicry in mice and a peptide in *S. aureus* that induces anti-MPO T cell immunity [11]. There is no current literature on a relationship between *S. aureus*, the oral microbiome, and strawberry gingivitis; however, with the case finding of strawberry gingivitis as a presenting symptom in GPA relapse, more research in the future could be beneficial to the early diagnosis and treatment of relapses for GPA. 

To treat GPA, patients are classified by either limited or severe extent of involvement and then are treated with immunosuppressive agents in two phases, an induction and maintenance phase [3]. The choice of therapy is determined by patient and physician preference and the associated adverse effects. Limited disease does not pose threats to life or organ systems and is most commonly treated with methotrexate in combination with glucocorticoids [3]. Severe disease includes all organ- or life-threatening diseases such as pulmonary hemorrhage, progressive neuropathy, and active glomerulonephritis and is thereby most commonly treated with cyclophosphamide and glucocorticoids [3]. After remission has been induced, low-risk patients can transition to maintenance therapy to avoid relapses for 12-36 months, and for high-risk patients, maintenance therapy is continued indefinitely [3]. The maintenance phase of treatment is commonly adjusted and changed with each patient as the rate of GPA symptom relapse is about 20% at 12 months and rises to 60% at 60 months [12]. Common maintenance therapies consist of low-dose glucocorticoids with azathioprine, rituximab, methotrexate, or mycophenolate [13]. Early recognition of disease relapse can aid physicians in the early modification of maintenance therapy and help limit the severity of relapsing symptoms, which has been the downfall of most physicians in the management of this chronic relapsing and remitting disease [14]. 

Gingival hyperplasia and oral manifestations are key findings for GPA, but many other diseases can present with similar manifestations [8]. Some of the differentials for the oral manifestation seen in this patient were drug-induced gingival hyperplasia, a retained foreign body in the gingiva causing gingival swelling and ulceration, oral ulceration, or gingival enlargement stemming from a granulomatous disease. Considering the patient's history and past diagnosis of GPA, it was relatively easier to determine that the oral lesion was strawberry gingivitis, and this was a unique initial presenting sign of GPA relapse.

## Conclusions

GPA is a chronic relapsing and remitting vasculitis that has multisystemic effects and can lead to morbid outcomes without prompt recognition and treatment. GPA has many characteristic relapsing manifestations that are common in the disease, but strawberry gingivitis as the initial sign of recurring GPA is a rare presentation that should be recognized by both physicians and oral health professionals to allow for early and effective treatment. The early identification of the manifestation may lead to modification of patient maintenance therapy, which may save the patient from the later debilitating and systemic symptoms seen in GPA.
